# Laparoscopic assisted trans-scrotal orchiopexy versus traditional orchiopexy for inguinal cryptorchidism: a retrospective study based on 154 patients

**DOI:** 10.1186/s12894-023-01244-3

**Published:** 2023-05-06

**Authors:** Qiang Guo, Yifei Zhang, Huajian Lai, WenWen Zhong, Jianguang Qiu, Dejuan Wang

**Affiliations:** Department of Urology, The Sixth Affiliated Hospital, Sun Yat-sen University, No 26 Yuancun Erheng Road, Guangzhou, 510655 Guangdong Province China

**Keywords:** Laparoscopic assisted trans-scrotal surgery, Inguinal surgery, Cryptorchidism, Patent processus vaginalis, Orchidopexy

## Abstract

**Background:**

The purpose of this study was to investigate the clinical effect of laparoscopic assisted trans-scrotal orchiopexy versus traditional orchiopexy for inguinal cryptorchidism.

**Methods:**

A retrospective analysis of cryptorchidism patients who were admitted to our hospital from July 2018 to July 2021. The patients were divided into the laparoscopic assisted trans-scrotal surgery group (n = 76) and the traditional surgery group (n = 78) according to the surgical method.

**Results:**

All patients were successfully operated. There was no significant difference in operation time between the laparoscopic assisted trans-scrotal group and the traditional group (*P*>0.05). Although there was no significant difference in the postoperative hospital stay between the two groups, the time of postoperative hospital stay of the laparoscopic assisted trans-scrotal surgery group was lower than that in the traditional surgery group (*P* = 0.062). Additionally, there was no significant difference in discharge rate on the first day after surgery between the two groups, but the discharge rate on the first day after surgery was more than 90% in both groups. In terms of postoperative complications, there were no cases of testicular retraction, testicular atrophy, inguinal hernia, or hydrocele that occurred in both groups. There was no significant difference in the incidence of scrotal hematoma between the two groups(*P*>0.05). Although there was no significant difference in the incidence of poor wound healing between the two groups(*P*>0.05), the incidence in the laparoscopic assisted trans-scrotal surgery group was lower than that in the traditional surgery group (2.6% vs. 6.4%).

**Conclusion:**

Laparoscopic assisted trans-scrotal surgery is as safe and effective method as traditional surgery for patients with inguinal cryptorchidism, and could also provide a good appearance.

## Background

Cryptorchidism is a common congenital malformation found in 1.1–45.3% of preterm and 1.0–4.6% of full-term male neonates [1]. In most cases, the testes spontaneously descend over time, but 1.0–2.0% of boys will still have cryptorchidism at six months of age [2]. Cryptorchidism is a recognized risk factor for testicular cancer, infertility/sub-fertility, and testicular injury [3, 4]. Early management of cryptorchidism is needed to avoid progressive degenerative changes in the testes and subsequent infertility.

It is generally accepted that inguinal orchiopexy is a classic method for inguinal undescended testes [5, 6], but inguinal orchidopexy may lead to inguinal scars and post-operative pain, which affects the patient’s appearance. With the development of medicine, minimally invasive surgery has become the mainstream surgical method. Laparoscopic and trans-crotal orchidopexy are favored by patients because of less trauma, less pain, faster recovery, and better cosmetic appearance [7–10]. However, for low or high cryptorchidism, laparoscopic orchiopexy or trans-scrotal orchiopexy alone has defects. Laparoscopic assisted scrotal orchiopexy seems to be a better choice [11]. This article retrospectively analyzed the clinical data and summarized the experience of laparoscopic assisted scrotal surgery for inguinal cryptorchidism.

## Methods

### Patients

This retrospective study analyzed the clinical data of 154 patients with inguinal cryptorchidism at our hospital between July 2018 and July 2021. Children with recurrent inguinal cryptorchidism after surgery in this study were excluded. The patients were divided into the laparoscopic assisted trans-scrotal surgery group (n = 76) and the traditional surgery group (n = 78) according to the surgical method.

The follow-up times are one week, three months, six months, and one year after the surgery. The positions and sizes of both testes are also evaluated by scrotal color Doppler ultrasonography at six months and one year after the operation. The following data were extracted from the electronic medical records: age, size of cryptorchid testis, side of cryptorchidism, operation time, duration of hospital stay, and post-operative complications.

### Laparoscopic assisted trans-scrotal orchiopexy

After anesthesia induction, the patient was in a supine position with slightly elevated hip. Routine disinfection of the surgical area and laying of the surgical sheet. The pneumoperitoneum (10mmHg) was established. A 5 mm trocar was placed in the umbilical area, and a 3 mm trocar was placed slightly below the umbilical margin. The affected spermatic cord blood vessel and the vas deferens are dissected until they are of adequate length. A trans-scrotal incision is subsequently made at the bottom of the affected scrotum. The testis is dragged into the scrotum through the Hesselbach’s triangle and the mobilized testicle needs to be placed in a sub-dartos pouch within the hemi-scrotum without any tension. High ligation of the patent processus vaginalis or inguinal inner ring (if existed) is achieved using an 18-gauge intravenous indwelling needle with a suture. The incision was closed by 5–0 absorbable sutures.

### Traditional orchiopexy

The traditional Orchiopexy via the trans-inguinal approach.

### Statistical analysis

All statistical analyses were performed using the IBM SPSS Statistics for Windows, Version 25.0 (Armonk, NY, USA). The continuous data were summarized using mean ± standard deviation (SD) or median (range) and analyzed using the student’s t-test and the Mann-Whitney U test, as appropriate. Categorical data were expressed as numbers and percentages and analyzed using the chi-square test. A *p* value < 0.05 was considered statistically significant.

## Results

The Baseline and post-operative characteristics of patients are shown in Table 1. There was no significant difference in age, size of cryptorchid testis, and side of cryptorchidism (*P*>0.05).

**Table 1:** Baseline, intraoperative and postoperative data of patients

Clinical data	Laparoscopic assisted trans-scrotal surgery group	Traditional surgery group	P value
Number	76	78	
Age (years)	3.22 ± 2.08	3.09 ± 2.71	0.261
Size of cryptorchid testis (mm)	7.40 ± 1.50	7.26 ± 1.54	0.214
Side of cryptorchidism			0.621
Unilateral	49	47	
Bilateral	27	31	
Patent processus vaginalis	20	21	0.895
Operative time (hours)			
Unilateral cryptorchidism	1.40 ± 0.56	1.38 ± 0.47	0.843
Bilateral cryptorchidism	1.72 ± 0.50	1.65 ± 0.59	0.631
Postoperative hospital stay (hours)	15.0 ± 8.09	18.2 ± 12.5	0.062
Discharge rate on the 1st day after surgery	71 (93.4%)	73 (93.6%)	0.966
Size of cryptorchid testis one year after surgery (mm)	14.8 ± 2.69	14.9 ± 2.88	0.896

All patients in the laparoscopic assisted trans-scrotal surgery group were successfully operated, and no patients were converted to the traditional surgery group. For patients of unilateral or bilateral cryptorchidism, there was no significant difference in operation time between the two groups (*P*>0.05). Although there was no significant difference in the postoperative hospital stay between the two groups, the time of postoperative hospital stay of the laparoscopic assisted trans-scrotal surgery group was lower than that in the traditional surgery group. Additionally, there was no significant difference in discharge rate on the first day after surgery between the two groups, but the discharge rate on the first day after surgery was more than 90% in both groups.

In terms of postoperative complications, there were no cases of testicular retraction, testicular atrophy, inguinal hernia, or hydrocele occurred in both groups. There was no significant difference in the incidence of scrotal hematoma between the two groups. Although there was no significant difference in the incidence of poor wound healing between the two groups, the incidence in the laparoscopic assisted trans-scrotal surgery group was lower than that in the traditional surgery group (2.6% vs. 6.4%).

All patients were followed up for more than one year, with a median follow-up time of 16 months. At one year after surgery follow-up, the size of cryptorchid testis was 14.8 ± 2.69 mm in the laparoscopic assisted trans-scrotal surgery group and 14.9 ± 3.03 in the traditional surgery group, indicating an improvement compared with that before surgery, but there was no significant difference in the two groups (*P* = 0.896).

## Discussion

Cryptorchidism is a common condition in boys [12]. The testes may continue to descend till six months of age after which the chances of spontaneous descent are very low. Children with undescended testis at six months of age should be operated before a year of age [2, 13–15]. Undescended testis is a well-known risk factor for testicular injury, infertility/sub-fertility, and testicular malignancy. Therefore, early management, regular follow-up, and assessment are necessary [16, 17].

Surgery is the main treatment for cryptorchidism. The traditional method is trans-inguinal orchidopexy which is effective, and suitable for inguinal testes. But this surgery has many disadvantages such as postoperative wound pain, and scars [18]. With the increasing demands of aesthetics, minimally invasive surgery has gradually become the mainstream of cryptorchidism surgery.

A meta-analysis compared the clinical effects of trans-scrotal and trans-inguinal orchidopexy and concluded that though the treatment effects were similar, trans-scrotal orchidopexy had less postoperative pain, better cosmetic results, and shorter operative time and hospital stay [9]. However, trans-scrotal surgery is only suitable for low inguinal cryptorchidism. laparoscopic surgery has been widely used due to the benefits of better visualization, and the simultaneous opportunity for exploring and ligating the patent processus vaginalis without cutting the outer inguinal ring. Also, it is possible to dissect the spermatic vessels and vas deferens under direct visualization and to preserve the blood supply of the testes, so that it can be brought down into the scrotum, tension-free [10, 18, 19]. However, laparoscopic surgery is not suitable in cases of low inguinal cryptorchidism. In this study, we combined laparoscopic and trans-scrotal surgery for laparoscopic assisted trans-scrotal surgery for inguinal cryptorchidism. Although, there was no significant difference in operation time and postoperative hospital stay between the laparoscopic assisted trans-scrotal group and the traditional group (*P*>0.05), the time of postoperative hospital stay of the laparoscopic assisted trans-scrotal surgery group was lower than that in the traditional surgery group (*P* = 0.062). It is believed that if the sample size is expanded, the difference between the two groups will become significant.

Patients with cryptorchidism had a high incidence of patent processus vaginalis. The patent processus vaginalis needs to be ligated proximally at the level of the internal ring because an unidentified or inadequately repaired patent processus vaginalis is an important factor leading to failure of orchidopexy [20, 21]. Furthermore, insufficient ligation of patent processus vaginalis may lead to hydrocele or inguinal hernia after the operation. In this study, we used an 18-gauge intravenous indwelling needle with a suture for ligating the patent processus vaginalis and there were no hydrocele or inguinal hernia occurred during the follow-up period in these patients.

Scrotal hematoma and poor wound healing were the two major complications in this study. Trans-scrotal surgery is easy to cause scrotal hematoma if the hemostasis is not complete, while inguinal surgery is prone to wound infection or poor wound healing due to the particularity of the surgical site. Therefore, attention should be paid to hemostasis during the trans-scrotal surgery and good disinfection, and nursing after inguinal surgery. At one year follow-up after the operation, there were no cases of testicular retraction, testicular atrophy, inguinal hernia or hydrocele occurred in both groups (Table 2), and furthermore, the improvement of the size of cryptorchid testis was similar, which indicated that the laparoscopic assisted trans-scrotal surgery and traditional surgery had good and similar clinical effects.

**Table 2:** Comparison of postoperative complications between the two groups

Item	Laparoscopic assisted trans-scrotal surgery group	Traditional surgery group	P value
Number	76	78	
Testicular retraction	0	0	
Testicular atrophy	0	0	
Inguinal hernia	0	0	
Hydrocele	0	0	
Scrotal hematoma	6 (7.9%)	4 (5.1%)	0.489
Poor wound healing	2 (2.6%)	5 (6.4%)	0.263

Our study has certain limitations. Firstly, it was a single-center retrospective study with small sample size. Secondly, our follow-up period was short. Well-designed randomized controlled trials with long follow-ups are required to study the efficacy of the laparoscopic assisted trans-scrotal surgery in inguinal cryptorchidism.

## Conclusion

Laparoscopic assisted trans-scrotal surgery is as safe and effective methods as traditional surgery for patients with inguinal cryptorchidism, and could also provide a good appearance.

## Data Availability

The datasets supporting the results of this article are included within the article and also the datasets used and/or analyzed during the current study are available from the corresponding author on reasonable request.
